# Insights into the Relationship Between the Gut Microbiome and Immune Checkpoint Inhibitors in Solid Tumors

**DOI:** 10.3390/cancers16244271

**Published:** 2024-12-23

**Authors:** Sona Ciernikova, Aneta Sevcikova, Maria Novisedlakova, Michal Mego

**Affiliations:** 1 Department of Genetics, Cancer Research Institute, Biomedical Research Center of the Slovak Academy of Sciences, Dubravska Cesta 9, 845 05 Bratislava, Slovakia; aneta.sevcikova@savba.sk; 2 Department of Oncology, Hospital Bory, Ivana Bukovčana 6118, 841 08 Bratislava, Slovakia; maria.novisedlakova@gmail.com; 3 2nd Department of Oncology, Faculty of Medicine, Comenius University, Bratislava and National Cancer Institute, Klenova 1, 833 10 Bratislava, Slovakia; misomego@gmail.com

**Keywords:** the gut microbiome, cancer treatment, immune checkpoint inhibitors, solid tumors, probiotics, fecal microbiota transplantation

## Abstract

Immunotherapy with immune checkpoint inhibitors represents a revolutionary approach to the treatment of solid tumors, including malignant melanoma, lung cancer, and gastrointestinal malignancies. Anti-CTLA-4 and anti-PD-1/PDL-1 therapies provide prolonged survival for cancer patients, but their efficacy and safety are highly variable. This review focuses on the crucial role of the gut microbiome in modulating the efficacy and toxicity of immune checkpoint blockade. Studies suggest that the composition of the gut microbiome may influence the response to immunotherapy, with specific bacterial strains able to promote an anti-tumor immune response. On the other hand, dysbiosis may increase the risk of adverse effects, such as immune-mediated colitis. Interventions aimed at modulating the microbiome, including the use of probiotics, prebiotics, fecal microbial transplantation, or dietary modifications, represent promising strategies to increase treatment efficacy and reduce toxicity. The combination of immunotherapy with the microbiome-based strategy opens up new possibilities for personalized treatment. In addition, factors such as physical activity and nutritional supplementation may indirectly influence the gut ecosystem and consequently improve treatment outcomes in refractory patients, leading to enhanced patient responses and prolonged survival.

## Simple Summary

Immune checkpoint inhibitors (ICIs) offer an innovative strategy for treating solid tumors by harnessing the host’s immune system to destroy cancer cells. Microbiota signatures show their potential in predicting the prognosis and modulating ICI efficacy in solid tumors. Advances in microbiome-based research have shown that microbiota modulation, specifically through fecal transplants from ICI responders to non-responders, can improve treatment outcomes. In addition, the impacts of probiotics, prebiotics, dietary interventions, and physical activity on the gut microbiome composition and improved response to ICI treatment continue to attract growing interest. Enhancing the gut microbiota balance could help reduce immune-related adverse events (irAEs) and positively influence patients’ overall health and quality of life.

## 1. Introduction

The gut microbiome is a complex community of microorganisms inhabiting the human gastrointestinal tract. It comprises bacteria, archaea, viruses, fungi, and protozoa, along with their collective genomes and metabolic potential [1,2,3,4]. Among these, bacteria are the most abundant, contributing significantly to overall health [5,6,7]. The composition of the gut microbiome is influenced by a broad spectrum of intrinsic and extrinsic factors, including genetics, type of birth, method of infant feeding, age, diet, use of antibiotics and other medications, physical activity, lifestyle, smoking, and numerous others [8,9,10,11].

A favorable gut microbiome composition is characterized by a broad microbial diversity and high colonization resistance [12,13,14,15]. It performs numerous vital roles, including protection against pathogens, and metabolizing complex carbohydrates into short-chain fatty acids (SCFAs), which serve as an energy source for gut epithelial cells and have anti-inflammatory properties [16,17,18,19,20]. Moreover, symbiotic microorganisms synthesize important vitamins and amino acids, control fat metabolism, produce small molecules that interact with the host environment, and shape the host’s immune system [21,22]. Gut homeostasis is mediated by the predominance of bacteria belonging to Firmicutes and *Bifidobacteriaceae*, while an increase in facultative anaerobic bacteria such as *Enterobacteriaceae* is a key indicator of a gut imbalance, known as dysbiosis, which can subsequently lead to the development of severe diseases, including cancer [23,24].

The gut microbiome influences the development and regulation of immune responses. It represents the defense against pathogens by producing bacteriocins and increasing colonization resistance, preventing the adverse effects of toxins, and playing a crucial role in maintaining the integrity of the gut barrier [25,26]. Intestinal microbial communities significantly influence immune homeostasis. A disruption of this balance is associated with inflammatory and immune-related diseases, as well as the response to immunotherapy [27,28,29,30]. The microbiome impacts immune cells through both direct and indirect effects at the cellular and molecular levels. SCFAs influence the fate of immune cells through direct epigenetic modification induced by changes in metabolism via histone deacetylase inhibition [31,32].

The immune system plays a crucial and complex role in the prevention of cancer initiation, progression, and metastasis [33,34]. Immunotherapy is a type of anti-cancer treatment that uses components of the immune system to fight cancer cells [35,36]. The principle of immunotherapy relies on either the direct manipulation of immune cell populations, primarily through immune checkpoint inhibitors (ICIs), or redirecting endogenous immune cells to tumors using target-specific proteins [33]. Preclinical murine models and clinical cohorts show that the gut microbiota significantly influences the efficacy and side effects of ICIs, and intestinal dysbiosis can lead to treatment resistance [37,38,39,40]. Specific bacteria can induce anti-tumor immune responses, thereby controlling the treatment response [41,42,43].

In this review, we summarize the latest insights into the relationship between the gut microbiome and immunotherapy with ICIs in solid cancers, focusing on skin cancer, thoracic malignancies, renal cell carcinoma (RCC), and gastrointestinal malignancies. Moreover, we discuss the impact of microbiota modulation by probiotics, prebiotics, fecal microbiota transplantation (FMT), diet, supplements, and physical activity on the efficacy of ICI treatment. Importantly, we provide a set of ongoing clinical trials investigating the association between the microbiome composition and the safety and efficacy of ICIs in solid malignancies.

## 2. Immune Checkpoint Therapy in Solid Tumors

Immunotherapy alone or combined with conventional treatments, such as radiotherapy and chemotherapy, has achieved considerable success as a standard treatment for many cancers [35,44]. Immune system checkpoints are molecules expressed on immune cells that modulate the activation level of the immune system. These checkpoints act as crucial regulators of the body’s immune response to prevent excessive or uncontrolled immune activity [45,46,47,48]. During the last few years, a variety of control molecules of the immune system have been identified, including programmed cell death protein 1 (PD-1)/programmed death-ligand 1 (PD-L1), cytotoxic T lymphocyte-associated protein 4 (CTLA-4), lymphocyte activating gene 3 (LAG-3), and T cell immunoglobulin and mucin domain-containing gene 3 (TIM-3), among others [45,49].

ICIs inhibit negative immune regulation to enhance immune activity against cancer cells [45,50,51,52]. Advanced and metastatic solid tumors remain incurable in most patients because conventional therapies targeting tumor cells usually fail to provide a durable response [35,44]. Cancer immunotherapy using ICIs has impacted the treatment of a wide range of human malignancies and offers long-term clinical responses and the possibility of a cure for many more patients with metastatic cancer [44,45,50,52].

From 2011, when the first ICI ipilimumab was approved by the Food and Drug Administration (FDA), to 2024, the indications for ICI therapy have significantly expanded across multiple solid tumors, not only for the treatment of metastatic and advanced disease but also for maintenance, neoadjuvant, and even adjuvant treatment of selected malignancies. ICIs are applicable in treating a broad spectrum of solid cancers, including melanoma, lung cancer, lymphoma, gastrointestinal and urogenital malignancies, breast cancer, and others, as a monotherapy, a combination of two ICIs, or a combination of ICIs and chemotherapeutic agents [45,53,54,55].

FDA has consecutively approved three distinct categories of ICIs: CTLA-4 inhibitors (ipilimumab and tremelimumab), PD-1 inhibitors (nivolumab, pembrolizumab, and cemiplimab), and PD-L1 inhibitors (atezolimumab, durvalumab, and avelumab) [35] with specific medical indications [56].

Ipilimumab is an FDA-approved ICI for treating unresectable or metastatic melanoma and for high-risk stage III melanoma [56].

Nivolumab gradually received approval for the treatment of melanoma, non-small cell lung cancer (NSCLC), RCC, classic Hodgkin lymphoma (cHL), head and neck squamous cell carcinoma (HNSCC), urothelial carcinoma (UC), patients with microsatellite instability-high (MSI-H) or mismatch-repair deficiency (dMMR) metastatic colorectal cancer, hepatocellular carcinoma (HCC), esophageal squamous cell carcinoma, gastric cancer, gastroesophageal junction cancer (GEJ), and esophageal adenocarcinoma [56].

Pembrolizumab has been approved for the treatment of NSCLC, refractory cHL UC, gastric or GEJ adenocarcinoma, cervical cancer, primary mediastinal B-cell lymphoma, HCC, Merkel cell carcinoma, RCC, HNSCC, small cell lung cancer (SCLC), esophageal and endometrial carcinoma, cutaneous squamous cell carcinoma, unresectable or metastatic TMB-high (≥10 mut/Mb) solid tumors, MSI-H or dMMR colorectal cancer, triple-negative breast cancer, GEJ carcinoma, biliary tract cancer, advanced bladder cancer, and malignant pleural mesothelioma [56].

Cemiplimab was approved for the treatment of cutaneous squamous cell carcinoma, advanced basal cell carcinoma, and NSCLC [56].

Durvalumab was approved for the treatment of NSCLC, SCLC, biliary tract cancer, and endometrial cancer [56]. Atezolizumab can be used for UC, NSCLC, SCLC, triple-negative breast cancer, melanoma, and alveolar soft part sarcoma [56]. Avelumab obtained FDA approval for Merkel cell carcinoma, UC, and RCC [56].

The use of ICIs for some of the above indications is determined by the extent of the disease, the previous treatment regimen, the presence of biomarkers (expression of PD-L1, CPS, TPS, MSI-H, dMMR, tumor mutational burden, EGFR, and ALK alterations), and the concomitant administration of a certain cytostatic regimen and biologic therapy.

Immune checkpoint therapy (ICT) may provide durable clinical responses and improve overall survival. However, only subsets of patients with specific tumor types respond to ICT. Most patients do not respond and some patients develop resistance to treatment after an initial response. In addition, ICIs can result in life-threatening toxicities known as immune-related adverse events (irAEs) [35,53]. This highlights the role of biomarkers, including TMB, PD-L1 expression, the microbiome, hypoxia, interferon-gamma (IFN-γ), and ECM in predicting responses to ICI-based immunotherapy [35]. Consequently, significant challenges remain, including understanding resistance pathways, optimizing patient selection, improving the management of irAEs, and identifying rational therapeutic combinations [53].

## 3. The Impact of the Gut Microbiome on Immunotherapy Efficacy and Treatment-Related Toxicity

**Figure 1:**
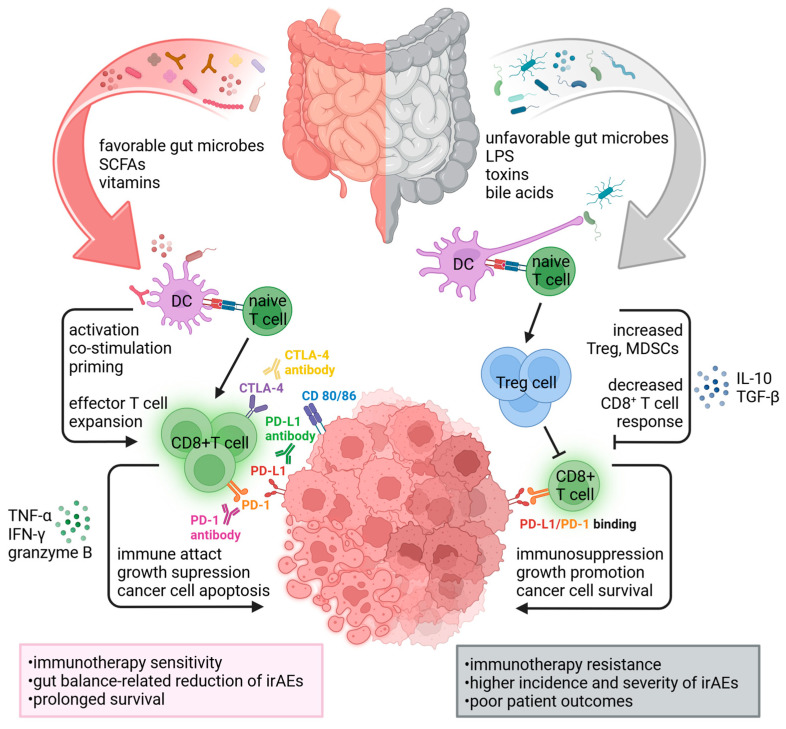
The associations between the gut microbiome composition, immune checkpoint inhibitors, and immune responses against cancer. A favorable gut microbiome and high bacterial diversity enhance the maturation of DCs and T cell priming, leading to the release of specific cytokines that drive the expansion of effector CD8+ T cells. Activation of tumor-specific CD8+ T cells and higher levels of serum IFN-γ, TNF-α, and granzyme B contribute to cancer cell death. Modulating the immune cell responses by commensal bacteria enhances tumor sensitivity to ICIs. In contrast, an unfavorable microbiome composition and decreased bacterial diversity are associated with elevated levels of Treg cells and MDSCs, leading to reduced amounts of cytotoxic CD8+ T cells and cancer immune evasion. Additionally, intestinal dysbiosis can create a tumor microenvironment less conducive to effective CD8+ T cell activity. Abbreviations: CTLA-4, cytotoxic T lymphocyte-associated protein 4; DC, dendritic cell; ICIs, immune checkpoint inhibitors; IFN-γ, interferon-gamma; IL-10, interleukin-10; MDSCs, myeloid-derived suppressor cells; PD-1, programmed cell death protein 1; PD-L1, programmed death-ligand 1; SCFAs, short-chain fatty acids; TGF-β, transforming growth factor-beta; TNF-α, tumor necrosis factor-alpha; Treg cell, regulatory T cell.

Immunotherapy engages the patient’s immune system to identify and eliminate tumor cells, representing a revolutionary approach to cancer treatment [57]. Due to its critical role in modulating immune system reactions, a close relationship between the microbiome composition and ICI treatment has been uncovered (Figure 1). Recently, a multi-cohort trans-kingdom analysis of 12 datasets revealed that the bacterial member *Faecalibacterium prausnitzii*, associated with ICI responders, correlated with longer progression-free survival (PFS) in melanoma patients. In NSCLC patients, *Candidatus gastranaerophilales* [CG.] bacterium MH-37 and *Clostridia* spp. were linked to an extended PFS. These findings suggest that identifying baseline gut bacterial biomarkers in the intestinal tract could serve as potential predictors of the ICI response [58]. Mounting evidence has documented the impact of microorganisms residing in the tumor microenvironment (TME) on immunotherapy efficacy by influencing immune responses either directly or through the production of microbiota-derived metabolites [59].

### 3.1. Malignant Melanoma and Cutaneous Squamous Cell Carcinoma

Melanoma patients exhibit different compositions of microbial communities relative to the ICI response status [60,61]. However, the results of microbiome analyses have shown inconsistency across various studies. Using selbal analysis, significant differences in microbial communities at the genus level were identified between responders and non-responders to ICIs. The multivariate selbal algorithm, which employs modeling and variable selection methods, pinpointed nine genera that were more abundant in non-responders, while eleven genera were more prevalent in responders. Additionally, univariate tests identified seven genera with significant differences between the two groups. These findings suggest that machine learning models could help uncover associations between the microbiome and immunotherapy, potentially leading to improved outcomes and quality of life for cancer patients [62]. Zakharevich et al. developed a catalog of metagenome-assembled genomes (MAGs) using fecal samples from melanoma patients, identifying 1416 bacterial metagenomes across 13 phyla. The list of MAG biomarkers highlighted the importance of *Bifidobacterium adolescentis*, *Bifidobacterium longum*, *Bifidobacterium bifidum*, and *Bifidobacterium angulatum*, which were associated with favorable immunotherapeutic outcomes [63].

Stool sample sequencing indicated an enrichment of *Ruminococcaceae* and *Faecalibacterium* in responders, while non-responders had higher levels of *Bacteroides thetaiotaomicron*, *Escherichia coli*, and *Anaerotruncus colihominis*. The presence of beneficial *Faecalibacterium* species was positively associated with increased levels of effector CD4+ and CD8+ T cells in the circulation, enhancing both anti-tumor and systemic immune responses [64]. Higher levels of Firmicutes in stool samples positively correlated with a favorable response to ICIs in melanoma patients, while the prevalence of the *Bacteroidales* family was observed in patients with a poor prognosis. Overall, microbial diversity appears to influence the treatment efficacy, with greater gut microbial diversity linked to better therapeutic outcomes [65]. Wind et al. identified 17 metabolic pathways that were differentially abundant in ICI-responding and non-responding metastatic melanoma patients. Although alpha diversity did not differ significantly between the two groups, 68 bacterial taxa were found to be differentially represented. Patients with *Streptococcus parasanguinis* in their gut microbiome showed longer overall survival (OS), and *Bacteroides massiliensis* was associated with prolonged PFS [66]. Another study documented that *Bifidobacterium adolescentis*, *Faecalibacterium prausnitzii*, and *Eubacterium rectale* in fecal samples may serve as potential biomarkers of the response to immunotherapy in melanoma patients [67].

The efficacy of CTLA-4 blockade in treating metastatic melanoma appears to be significantly influenced by the presence of certain *Bacteroides* species, particularly *Bacteroides fragilis*. These specific microbial populations might play a role in optimizing the therapeutic response [68]. An enrichment of *Eubacterium rectale*, observed in the gut microbiome of melanoma patients, correlated with higher anti-PD-1 efficacy and prolonged survival. In a mouse model, anti-PD-1 combined with *Eubacterium rectale* increased IFN-γ expression in CD4+/CD8+ T cells, indicating enhanced T cell activity. As shown, *Eubacterium rectale* boosted anti-PD-1 efficacy through NK cell activation [69]. In a study with mice bearing melanoma, the administration of the bacteria-derived metabolite desaminotyrosine (DAT) suppressed tumor growth and increased the response to CTLA-4 treatment [70]. This metabolite, derived from *Clostridium orbiscindens*, enhances IFN-I signaling [71], subsequently activating immune responses within the TME to promote tumor suppression.

Indole-3-carboxaldehyde (3-IAld) is a gut microbiome-derived metabolite contributing to the function of the epithelial barrier and gut homeostasis via aryl hydrocarbon receptor (AhR). The administration of 3-IAld before immunotherapy improved the survival of mice with ICI-induced colitis. The protective effect of 3-IAld was responsible for decreased levels of tumor necrosis factor-alpha (TNF-α), IL-1β, and IL-17A. However, another animal experiment showed that 3-IAld had no impact on the efficacy of CTLA-4 blockade or tumor growth in melanoma-bearing mice [72].

A study involving 94 melanoma patients treated with anti-PD-1 revealed changes in the gut microbiome composition, neutrophil-to-lymphocyte ratio (NLR), and the occurrence of irAEs. Patients with a high NLR exhibited reduced survival and a microbiome rich in gram-negative bacteria, whereas those with a low NLR experienced longer survival. According to the findings, Actinobacteria and Firmicutes (e.g., *Lachnospiraceae* and *Ruminococcaceae*) were predominant in the gut of patients responding to treatment. At the same time, Bacteroidetes, including *Prevotellaceae*, *Porphyromonadaceae*, *Rikenellaceae*, and *Bacteroidaceae*, were more abundant in the stool samples from the non-responders [73].

A study of patients with cutaneous squamous cell carcinoma comparing responders and non-responders to PD-1 blockade with cemiplimab showed no differences in intratumoral microbes (e.g., the analysis of the bacteriome, virome, and mycobiome) between the studied groups [74].

Ongoing clinical trials evaluating the link between the gut microbiome and ICI treatment in patients with melanoma and cutaneous squamous cell carcinoma are placed in Table 1.

### 3.2. Thoracic Cancers and Renal Cell Carcinoma

*Akkermansia muciniphila* was linked to improved survival in patients with advanced NSCLC and RCC treated with PD-1 blockers [75]. Salgia et al. also observed that RCC patients who experienced clinical benefits from ICI treatment exhibited an increased abundance of *Akkermansia muciniphila*. Moreover, a higher microbial diversity was significantly linked to improved efficacy of ICI treatment (*p* = 0.001) [76]. On the other hand, patients who received antibiotics a month before treatment showed reduced OS and PFS, suggesting that antibiotic-induced gut dysbiosis might impair ICI efficacy. A greater diversity correlated with more favorable therapeutic outcomes and delayed disease progression [75,77].

Zeng et al. described a variation in the abundance of specific bacterial species among patients with advanced thoracic malignancies treated with anti-PD-1. Beneficial microorganisms, such as *Lachnospiraceae*, *Ruminococcaceae*, *Agathobacter*, and *Faecalibacterium*, which are known to enhance ICI efficacy, declined in patients with secondary resistance. Additionally, butyrate-producing bacteria such as *Roseburia*, *Ruminoccocus*, and *Clostridium* were found in lower quantities upon resistance development. Patients experiencing irAEs showed a marked reduction in Bacteroides, with levels returning to normal after remission [78]. An enrichment of *Ruminococcus* was observed in Chinese patients with NSCLC who did not respond to nivolumab. On the other hand, the bacterial species *Alistipes putredinis*, *Bifidobacterium longum*, and *Prevotella copri* had increased abundance in responding patients. Additionally, the frequencies of peripheral memory CD8+ T cells and NK cells were positively correlated with a higher alpha diversity index among responders. These findings underscore the connection between the microbiome, immune system, and therapeutic response [79]. Lung cancer patients who responded positively to PD-1/PD-L1 blockade showed significantly higher levels of butyrate-producing genera, including *Faecalibacterium*, *Ruminococcus*, *Roseburia*, and *Anaerobutyricum*. At lower taxonomic levels, *Anaerobutyricum hallii*, *Blautia faecis*, *Coprococcus catus*, *Eubacterium ramulus*, and *Faecalibacterium prausnitzii* were increased in ICI-responding patients. In accordance, a metabolome analysis revealed lower levels of butyrate/isobutyrate in patients who experienced immunotherapy resistance. The unfavorable bacterium *Prevotella copri* was prevalent in patients with irAEs. Meanwhile, other bacterial species, including *Akkermansia muciniphila*, *Bacteroides stercoris*, *Roseburia faecis*, *Roseburia intestinalis*, and *Lactobacillus mucosae*, were found to be reduced in these patients [80]. In patients with NSCLC, a pre-treatment intestinal composition that was enriched with favorable *Akkermansia* and had decreased levels of pro-invasive microbial taxa correlated with the response to treatment with nivolumab combined with chemotherapy or a combination of ipilimumab with nivolumab and chemotherapy [81].

A metabolomic study revealed that increased lactate levels after ICI administration correlated with poor NSCLC patient outcomes. Conversely, elevated levels of specific bile acids, glycochenodeoxycholic acid, and taurolithocholic acid were associated with patient’s therapeutic benefit and improved survival. As found, taurolithocholic acid enhanced T cells’ anti-tumor response [82]. In patients with advanced-stage SCLC who underwent PD-L1 blockade, alpha diversity was higher in responders after treatment. Additionally, the authors observed changes in the composition of bacterial communities following ICI therapy. Responders showed a dominance of bacterial species such as *Faecalibacterium*, *Clostridium sensu stricto 1*, and *Ruminococcus torques*, while *Dubosiella* and *Coriobacteriaceae UCG-002* correlated with poor outcomes. A metabolite analysis revealed that microbiome-derived SCFAs were significantly enriched in post-treatment samples from responders [83]. Identifying microbiota-associated biomarkers represents a crucial step in the development of personalized treatment strategies for NSCLC patients [84].

Ongoing clinical trials evaluating the link between the gut microbiome and ICI treatment in patients with thoracic malignancies and RCC are placed in Table 2.

### 3.3. Gatrointestinal Malignancies

*Coprobacillus cateniformis* and *Erysipelatoclostridium ramosu* reduce PD-L2 expression on dendritic cells (DCs) in MC38 colon carcinoma cells. PD-L2, a binding partner of PD-1, can bind RGMb, whose reduced expression contributes to enhanced anti-tumor immunity. RGMb deletion in T cells, but not in macrophages or granulocytes, improved therapeutic responses in antibiotic-treated mice receiving PD-L1-based immunotherapy. Combining PD-1 blockade with blocking PD-L2/RGMb interactions may overcome microbiome-dependent resistance compared to PD-1 monotherapy, offering novel immunotherapy strategies [85]. Magahis et al. observed that *Helicobacter pylori* infection was linked to shorter OS and PFS in ICI-treated patients with advanced gastric cancer. The majority of these infected patients were non-white, younger, and Hispanic compared to non-infected subjects. Thus, detecting *Helicobacter pylori* infection through stool antigen tests might predict future immune responsiveness [86]. A recent longitudinal prospective cohort study involving patients with resectable esophageal carcinoma documented the link between lower alpha diversity in feces during ICI treatment and a poor response. Notably, higher baseline duodenal alpha diversity positively correlated with longer PFS. Baseline tumor microbiomes characterized by a high abundance of *Fusobacterium* and a lower presence of *Streptococcus* were related to shorter patient survival [87].

Ongoing clinical trials evaluating the link between the gut microbiome and ICI treatment in patients with gastrointestinal malignancies are placed in Table 3.

**Table 2:** List of ongoing clinical trials characterizing the gut microbiome composition in the context of the immunotherapy response, focusing on patients with thoracic malignancies and RCC. Moreover, several trials evaluated the relationship between sensitivity vs. resistance to PD-1, PD-L1, or CTLA-4 blockade and the gut microbiome not only in patients with lung cancer and RCC but also in patients with other mentioned malignancies (according to https://ClinicalTrials.gov/, accessed on 24 November 2024).

Study	Study Type	Malignancy	Purpose	Subjects (n)	Intervention/ Treatment	Study Status
NCT06630429	An observational prospective study	NSCLC/ RCC	To analyze stool samples for revealing the gut microbiome composition and determine circulating IL-6, other cytokine, glucocorticoid, and ketone levels	100 participants	NSCLC patients will receive ICIs alone or in combination with chemotherapy. RCC patients will be treated with nivolumab and ipilimumab.	Recruiting
NCT04913311	An observational prospective study	NSCLC	To define the gut microbial composition of fecal samples and identify predictive markers for therapy-related adverse events	150 participants	Subjects will receive standard-of-care treatment consisting of chemoradiation from the baseline up to week 10 and ICIs from weeks 10–62.	Recruiting
NCT03168464	An interventional Phase I and Phase II open-label study with single-group assignment	NSCLC	To investigate the associations between the overall response rate and gut microbiome alterations	15 participants	Radiotherapy will be directed to one lesion during the first immunotherapy with ipilimumab. On day 22, the combined treatment of ipilimumab + nivolumab will start and continue until progression.	Terminated (slow accrual)
NCT04013542	An interventional Phase I open-label study with single-group assignment	NSCLC	To correlate the gut microbiome with the response outcomes, PFS, and OS	21 participants	Participants will receive nivolumab and ipilimumab on day 1. Within 1 day of starting ICIs, patients will also undergo radiotherapy 5 days a week over 6–7 weeks in the absence of cancer progression or unacceptable toxicity.	Active, not recruiting
NCT04636775	An observational prospective study	NSCLC (stage IV)	To determine microbiome changes in responders vs. non-responders and assess the differences in microbial communities between patients with and without adverse events	48 participants	The clinical trial will include immunotherapy-naïve patients who will receive PD-1/PD-L1 blockers.	Recruiting
NCT05000710	An interventional Phase II open-label study with single-group assignment	Metastatic/ advanced NSCLC	To study stool samples for the exploration of predictive microbial biomarkers of clinical efficacy and to identify immune response signatures	29 participants	The treatment will consist of durvalumab + tremelimumab. After 21 days, 11 fractions of radiotherapy will be administrated to metastatic or primary lesions.	Recruiting
NCT05487859	An interventional Phase II open-label study with single group assignment	Advanced RCC	To evaluate the gut microbiome composition	24 participants	Therapy will include acarbose in combination with ICIs based on standard-of-care therapy.	Not yet recruiting
NCT04090710	An interventional Phase II randomized open-label study with parallel assignment	Metastatic RCC	To characterize the baseline gut microbiome and changes during treatment	66 participants	Ipilimumab combined with nivolumab will be administered alone or with cytoreductive stereotactic body radiation therapy.	Active, not recruiting
NCT04291755	An observational prospective case-only study	NSCLC/ CRC	To develop a sample bank for cancer patients for the characterization of the impact of the gut microbiome on the response to treatment	100 participants	All patients will be administered ICIs, including but not limited to pembrolizumab, nivolumab, ipilimumab, and atlizumab.	Recruiting
NCT04682327	An observational prospective case–control study	NSCLC	To assess the differences in the gut microbial diversity and composition between responders vs. non-responders and define the incidence of therapy-related adverse events	50 participants	The treatment will consist of PD-1/PD-L1 blockade.	Unknown status
NCT04063501	An observational prospective study	NSCLC	To study the gut microbiome composition for the prediction of subjects’ responses by a longitudinal assessment of PFS	30 participants	Analyzed subjects will adhere to PD-1 blockade, either as monotherapy or combined with chemotherapy.	Recruiting
NCT06321640	An observational study	Lung cancer/ breast cancer/ head and neck cancer/ CRC/ melanoma/ UC	To reveal the occurrence of specific microbial signatures associated with sensitivity, primary/secondary resistance, and therapy toxicity	265 participants	The clinical intervention will include ICIs.	Recruiting
NCT05037825	An observational prospective study	Malignant melanoma/NSCLC/ RCC/breast cancer	To investigate changes in the relative abundance of microbial taxa at baseline and 2 time points	800 participants	Treatment will consist of a single agent or combination with another ICIs or other treatment agents or modalities.	Recruiting
NCT04107168	An observational prospective study	Melanoma/renal cancer/lung cancer	To study the roles of gut microbiome signatures in PFS and OS predictions and correlations with irAEs. The clinical trial also aims to compare the pre-treatment microbiome composition with the therapeutic response.	1800 participants	Patients will receive monotherapy (nivolumab/pembrolizumab/durvalumab or atezolizumab) and combined immunotherapies (nivolumab + ipilimumab) with/without chemotherapy. One cohort will receive PD-L1 blockade with kinase inhibitors.	Recruiting

Abbreviations: CRC, colorectal cancer; ICIs, immune checkpoint inhibitors; IL-6, interleukin-6; NSCLC, non-small cell lung cancer; OS, overall survival; PD-1, programmed cell death protein 1; PD-L1, programmed death-ligand 1; PFS, progression-free survival; RCC, renal cell carcinoma; UC, urothelial carcinoma.

**Table 3:** List of ongoing clinical trials characterizing the gut microbiome composition in the context of the immunotherapy response, focusing on patients with gastrointestinal malignancies. Moreover, several trials evaluating the relationship between sensitivity vs. resistance to PD-1, PD-L1, or CTLA-4 blockade and the gut microbiome in patients with other malignancies are included (according to https://ClinicalTrials.gov/, accessed on 24 November 2024).

Study	Study Type	Conditions/ Health-Related Issues	Purpose	Subjects (n)	Intervention/ Treatment	Study Status
NCT04196465	An interventional Phase II single-arm open-label study with single-group assignment	Gastric cancer/ hepatocellular carcinoma/ esophageal cancer	To identify predictive and/or prognostic microbial biomarkers in stool samples	48 participants	Patients will be treated with two cycles of neoadjuvant PD-L1.	Recruiting
NCT05845450	An interventional non-randomized single-arm open-label study with single-group assignment	CRC	To investigate microbial communities in stool samples and the impact of the microbiome on patient outcomes	112 participants	Based on the pre-specified molecular profile, participants will be treated with CTLA-4 (botensilimab) and/or PD-1 (balstilimab) blockade.	Recruiting
NCT04744649	An interventional Phase II randomized open-label study with parallel assignment	Gastric cancer	To assess the differences in the gut microbial composition between patients who are sensitive or resistant to immunotherapy.	110 participants	One group will receive oxaliplatin + capecitabine or oxaliplatin+S-1. Another group will be treated with PD-1 blockade (JS001) + oxaliplatin + capecitabine or oxaliplatin+S-1.	Recruiting
NCT05177133	An interventional Phase II open-label study with single-group assignment	Gastric cancer/esophageal cancer	To characterize the gut microbiome as a potential biomarker for the therapeutic response	25 participants	Subjects will be treated with PD-1 blockade (retifanlimab) every 4 weeks and capecitabine, and oxaliplatin every 3 weeks.	Recruiting
NCT05199649	An interventional study	Esophageal cancer	To measure the differences in the gut microbiome composition and reveal gut microorganisms and metabolic markers as predictors of therapy efficacy	30 participants	PD-1 inhibition with sintilimab combined with chemotherapy will be administered simultaneously every 3 weeks.	Unknown status
NCT02960282	An observational case-only prospective study	CRC (stage IV)	To determine the composition of the gut microbiome and gene and protein expression levels, and compare the presence of bacterial species with the tumor response	21 participants	Patients will be treated with chemotherapy or ICIs (pembrolizumab or another PD-1/PD-L1 antibody).	Terminated (Slow accrual)
NCT05635149	An observational prospective study	CRC	To explore the relationship between the gut microbiome and therapy efficacy by sequencing stool samples	100 participants	One cohort will receive fruquintinib and PD-1 blockade + radiotherapy, and another will receive fruquintinib and PD-1 blockade.	Unknown status
NCT05462496	An interventional Phase II open-label study	Pancreatic cancer	To analyze changes in the tumor microenvironment following the depletion of the microbiome by antibiotics and PD-1 blockade and define a correlation with the tumor response	25 participants	Participants will receive antibiotics with pembrolizumab following chemotherapy.	Recruiting
NCT06094140	An interventional Phase II open-label study with single-group assignment	Pancreatic cancer	To monitor the gut microbial structure during neoadjuvant therapy and detect bacteria in tumor samples using fluorescent in situ hybridization	20 participants	All enrolled patients will receive mFOLFIRINOX for 6 cycles and durvalumab delivered for 3 cycles for resectable tumors. Patients will undergo restaging and surgical resection.	Recruiting
NCT05440864	An interventional Phase II open-label study	Hepatocellular carcinoma	To assess the gut microbiome before and after durvalumab and tremelimumab treatment and in patients on adjuvant durvalumab	28 participants	Treatment will consist of CTLA-4- blockade (tremelimumab) combined with PD-L1 inhibition (durvalumab) preoperatively, followed by adjuvant durvalumab.	Recruiting
NCT04291755	An observational prospective case-only study	NSCLC/ CRC	To develop a sample bank for cancer patients for the characterization of the impact of the gut microbiome on the response to treatment	100 participants	All patients will be administered ICIs, including but not limited to pembrolizumab, nivolumab, ipilimumab, and atlizumab.	Recruiting
NCT06321640	An observational study	Lung cancer/ breast cancer/ head and neck cancer/ CRC/melanoma/ UC	To reveal the occurrence of specific microbial signatures associated with sensitivity, primary/secondary resistance, and therapy toxicity	265 participants	The clinical intervention will include ICIs.	Recruiting

Abbreviations: CRC, colorectal cancer; CTLA-4; cytotoxic T lymphocyte-associated protein 4; ICIs, immune checkpoint inhibitors; PD-1, programmed cell death protein 1; PD-L1, programmed death-ligand 1; UC, urothelial carcinoma.

## 4. Microbiota Modulation and Immune Checkpoint Inhibitors

Various approaches exist to modify the gut microbiome and restore its beneficial composition [88]. Probiotics, prebiotics, and FMT, along with diet and physical activity, represent the most frequent modulators of the gut microbial communities in cancer patients. A favorable gut microbiome not only enhances the patient’s systemic immunity but also improves the clinical response to cancer treatment and reduces the incidence of treatment-related adverse events [89,90]. Gut microbiota modulation in immunocompromised cancer patients raises safety concerns [91]. Results from clinical studies and meta-analyses, including studies with large patient cohorts, documented the low risks of infectious complications following probiotic supplementation [13,92]. Current studies focused on FMT indicate the safety of this approach in cancer patients; however, long-term research is still needed for its routine use in clinical practice.

### 4.1. Probiotics and Prebiotics

Modulating the microbiota with beneficial probiotic microorganisms appears to be an efficient way to enhance the efficacy of immunotherapy. In an animal study, oral supplementation with *Enterococcus faecium* and *Enterococcus faecalis* before melanoma cell injection and anti-PD-L1 treatment led to a significantly reduced tumor size in experimental mice compared to those treated with anti-PD-L1 alone [93]. Immunotherapeutically active enterococci, including *Enterococcus durans*, *Enterococcus hirae*, and *Enterococcus mundtii*, possess a specific peptidoglycan composition, immunomodulatory activity, and remodeling capability due to secreted peptidoglycan hydrolase containing antigen A (SagA) and NlpC/p60 endopeptidase that preferentially hydrolyze cross-linked peptidoglycan fragments. This hydrolysis results in smaller muropeptides that more effectively activate the NOD2-binding oligomerization domain-containing protein 2, supporting immunotherapy and enhancing intestinal barrier function [94]. Bacteria expressing SagA improve anti-PD-1 and anti-CTLA-4 therapy effectiveness in colorectal cancer and fibrosarcoma models, fostering an adaptive immune response [93].

The administration of *Lactobacillus johnsonii* or the tryptophan-derived metabolite indole-3-propionic acid (IPA) has been shown to improve the response to CD8+ T cell-driven αPD-1 immunotherapy [95]. This modulation enhanced ICI efficacy by developing progenitor-exhausted CD8+ T cells across multiple cancer types, including melanoma, breast cancer, and colorectal malignancies [95]. An in vivo study involving animals with bladder tumors and melanoma demonstrated the positive impact of *Bifidobacterium* species on boosting anti-tumor immunity. Combining the oral gavage of a probiotic cocktail containing *Bifidobacterium breve* and *Bifidobacterium longum* and CPI treatment slowed tumor growth in mice with an unfavorable microbial composition [96]. The results of a meta-analysis involving 1123 NSCLC patients highlighted a trend toward using probiotics alongside CPI treatment. As documented, patients receiving CPIs together with probiotics experienced improvements in both PFS and OS [97].

### 4.2. Fecal Microbiota Transplantation

FMT is the process of collecting, preparing, and transferring stool from healthy donors to a recipient’s gastrointestinal tract, aiming to restore the gut balance and microbial diversity [98,99,100]. Strict donor selection, timing, frequency, and delivery methods are essential for the success of FMT [13,59,101,102,103]. Donors, ideally from centralized stool banks, must undergo rigorous screening for microbiota-influencing risk factors. FMT can be efficiently delivered via the upper gastrointestinal tract using oral capsules and endoscopy or via the lower gastrointestinal tract through colonoscopy or rectal tubes [104].

Fecal transfer from healthy individuals to colorectal tumor-bearing mice enhanced anti-tumor immunity and disease suppression by increasing the infiltration of immune cells (CD8+ and CD4+ T cells and NK cells) targeting tumor cells. FMT also reduced excessive gut inflammation by modulating cytokine expression, lowering the levels of pro-inflammatory cytokines such as IL-1a, IL-6, IL-12, and IL-17a, and increasing IL-10 levels [105].

An increasing number of clinical studies are investigating the efficacy of FMT in oncology patients, with the primary objectives of preventing and reducing treatment-related toxicity while enhancing therapeutic efficacy [106,107,108,109]. Notably, FMT is largely being explored as a supportive therapy to enhance immunotherapy efficacy [110,111,112,113]. The majority of metastatic melanoma patients treated with PD-1 blockade do not achieve a durable response. However, FMT has a positive effect in vivo and on refractory metastatic melanoma patients who had previously failed to respond to therapy. Gopalakrishnan et al. demonstrated that mice receiving FMT from melanoma patients who responded to anti-PD-1 immunotherapy exhibited significantly reduced tumor growth and improved treatment outcomes compared to mice receiving fecal transplants from non-responding patients [64]. According to the findings of Youngster et al., three out of five recruited patients obtained a partial or complete response to reinduced PD-1 blockade and the level of infiltrated intratumoral CD8+ T cells was increased after FMT. Moreover, 16S rRNA gene sequencing revealed a higher abundance of *Paraprevotellaceae* and a decrease in *Betaproteobacteria* following FMT [114]. A case report demonstrated that a single course of FMT from a responding donor with metastatic melanoma restored the pembrolizumab response followed by a reduced cancer burden in a recipient with refractory metastatic melanoma who had previously failed to respond to immunotherapy [115].

Matson et al. reported that the fecal microbiota of metastatic melanoma patients who responded to PD-1 blockade contained higher abundances of *Enterococcus faecium*, *Collinsella aerofaciens*, *Bifidobacterium adolescentis*, *Bifidobacterium longum*, *Klebsiella pneumoniae*, *Veillonella parvula*, and *Parabacteroides merdae*. In contrast, *Ruminococcus obeum* and *Roseburia intestinalis* dominated in non-responding patients. Fecal transplantation from non-responding patients into melanoma-bearing mice resulted in accelerated tumor growth and a lack of response to PD-1 blockade. Conversely, mice receiving fecal microbiota from responding patients exhibited slower tumor progression and a more robust therapeutic effect of PD-1 blockade [116].

Baruch et al. performed a fecal transfer from two metastatic melanoma patients responding to PD-1 blockade to recipients who had experienced disease progression after immunotherapy. The authors observed a renewed clinical response to nivolumab in three recipients after FMT. Donor stool samples contained bacteria from the *Lachnospiraceae*, *Veillonellaceae*, and *Ruminococcaceae families* [117]. Another study confirmed the clinical benefit of FMT in improving the immunotherapeutic response in six out of fifteen advanced melanoma patients with ICI-resistant disease who received transplants from responding patients. After FMT, recipients showed enhanced CD8+ T cell activity and low levels of immunosuppressive IL-8-producing myeloid cells. The recipients’ gut microbiomes shifted to resemble the donors’ profile, with notable increases in the abundances of *Lachnospiraceae*, *Ruminococcaceae*, *Bifidobacteriaceae*, and *Coriobacteriaceae*, together with a decrease in members of the Bacteroidetes phylum [118].

### 4.3. Diet, Supplements, and Physical Activity

Diet is a major factor influencing the structure of the gut microbiome. The influence of dietary patterns and geographic factors on cancer treatment efficacy is becoming a significant area of cancer research [119,120,121,122,123,124,125]. Simpson et al. conducted an integrated analysis involving melanoma patients treated with ICIs in the United States, as well as high-risk resectable metastatic melanoma patients from Australia and the Netherlands. The study found a greater abundance of the family *Ruminococcaceae* in responders, while *Bacteroidaceae* showed higher levels in non-responders. At baseline, lower intake of omega-3 fatty acids, reduced dietary fiber intake, and elevated C-reactive protein levels were linked to a poor therapeutic response. The authors also observed that geography influenced irAE-related microbial signatures across independent patient cohorts [126].

Patients receiving ICI treatment had an improved PFS after fiber was incorporated into their regimen. The results reported a 30% decrease in the risk of disease progression or mortality with every additional 5 g of fiber consumed. The most notable outcomes were observed in melanoma patients who consumed dietary fiber without additional probiotic supplementation [127]. Studies confirmed that vitamin B5, produced by gut microbes, exerts an immunostimulatory effect in the context of immunotherapy [128]. Bourgin et al. observed that supplementation with vitamin B5 elevated the efficacy of PD-L1 blockade in vivo. Furthermore, plasma samples from melanoma patients demonstrated a positive correlation between vitamin B5 levels and the immunotherapy response [129]. A combination of CpG treatment with the bacterial metabolite inosine, produced by *Bifidobacterium pseudolongum*, enhanced CTLA-4 efficacy in mice with melanoma and bladder tumors [130].

## 5. Conclusions and Future Directions

In the coming years, microbiome analyses are expected to become an integral part of clinical practice in cancer care. Promising results from preclinical and clinical research underscore the crucial role of the gut microbiome in modulating the efficacy of ICIs in solid tumors, with specific microbial profiles correlating to better or worse clinical outcomes. According to numerous findings, the abundances of *Akkermansia muciniphila*, *Faecalibacterium*, and *Bacteroides* correlate positively with an improved ICI treatment response. Further research is needed to identify and validate robust and reliable microbial biomarkers that could predict treatment outcomes.

Gut dysbiosis has been increasingly linked to immune system modulation and could potentially serve as a predictive marker for immunotherapy resistance. The administration of broad-spectrum antibiotics close to or during ICI treatment was found to reduce the efficacy of ICI therapy by disrupting the gut microbial diversity and impairing immune activation, which is essential for targeting and eradicating tumor cells. Optimizing the selection, timing, and duration of antibiotic treatment could provide sufficient time for microbiome recovery, potentially enhancing clinical outcomes for patients receiving ICI treatment. Therefore, future studies should focus on developing evidence-based guidelines for antibiotic administration during ICI therapy.

More in-depth analyses of the relationship between the gut microbiota and ICI resistance could lead to the development of non-invasive diagnostic tests that allow clinicians to personalize treatment strategies for cancer patients. However, confounding factors like the collection methods, sample size, study duration, genetics, geography, diet, and medication use make it difficult to establish a universal microbiome-based signature for predicting the response to immunotherapy. To address these challenges, future studies should focus on standardizing methodologies and investigating the optimal combination of beneficial gut microbiota characteristics in larger cohorts with different genetic backgrounds and dietary habits. Additionally, a metabolomic analysis revealed several connections between microbiota-derived metabolites and the response to ICIs. In this context, mounting evidence documented that microbiota modulation can positively enhance the anti-tumor immune response in patients, thus reducing tumor resistance to therapy.

Despite the numerous advancements, many questions remain unresolved regarding the proper administration, kinetics, durability, and efficacy of gut microbiome modulations. It is still unknown how to compare and combine different therapy modalities. Additional comprehensive research is crucial to fully understand the complex relationships between immune checkpoint blockade and the microbiome, as well as to determine the most efficient microbiome-based approaches for microbiota modification. A deeper understanding of the mechanisms underlying the microbiome composition and its interactions with the host’s immune system is essential for incorporating a microbiome-based approach into routine clinical practice and developing more targeted therapies for different types of solid tumors.

## Figures and Tables

**Table 1:** List of ongoing clinical trials characterizing the gut microbiome composition in the context of the immunotherapy response, focusing on patients with melanoma and cutaneous squamous cell carcinoma. Several trials evaluating the relationship between sensitivity vs. resistance to PD-1, PD-L1, or CTLA-4 blockade and the gut microbiome in patients with other malignancies are also included (according to https://ClinicalTrials.gov/, accessed on 24 November 2024).

Study	Study Type	Malignancy	Purpose	Subjects (n)	Intervention/ Treatment	Study Status
NCT05878977	An interventional open-label study with single-group assignment	Metastatic melanoma	To assess whether the gut microbiome composition and exosomal mRNA expression of PD-L1 and IFN-γ might predict the response to PD-1 and CTLA-4 blockade	150 participants	Patients will be treated with ICIs, and the dosage, frequency, and duration will be determined.	Recruiting
NCT04107168	An observational prospective study	Melanoma/ renal cancer/ lung cancer	To study the role of gut microbiome signatures in PFS and OS predictions and correlations with irAEs. The clinical trial also aims to compare the pre-treatment microbiome composition with the therapeutic response.	1800 participants	Patients will receive monotherapy (nivolumab/pembrolizumab/durvalumab or atezolizumab) and combined immunotherapies (nivolumab + ipilimumab) with/without chemotherapy. One cohort will receive PD-L1 blockers with kinase inhibitors.	Recruiting
NCT02858921	An interventional Phase 2 randomized open-label study with parallel assignment	Resectable melanoma	To correlate the composition of gut microbial communities with the ICI response and characterize patients’ dietary habits	60 participants	The treatment will consist of dabrafenib administered orally twice a day, trametinib administered orally once a day for 1 week, and/or pembrolizumab.	Active, not recruiting
NCT04207086	An interventional Phase II open-label study with single-group assignment	Melanoma (stage III)	To analyze the gut microbiome composition in baseline samples, prior to surgery at week 6, week 24, and at relapse, and evaluate microbiome associations with the ICI response	20 participants	Analyzed subjects will adhere to pembrolizumab and lenvatinib for 6 weeks, followed by surgery and then pembrolizumab alone for 46 weeks.	Active, not recruiting
NCT05545969	An interventional Phase II multicenter open-label study with single-group assignment	Mucosal melanoma	To determine the impact of the gut microbiome on response outcomes	0 participants	Patients will be treated with pembrolizumab and lenvatinib for 6 weeks, atezolizumab as monotherapy followed by surgery, and then pembrolizumab alone for 46 weeks	Withdrawn
NCT05418972	An interventional Phase II open-label study with single-group assignment	Cutaneous melanoma (stage III)	To correlate bacterial diversity and abundance with the immunotherapy response and incidence of treatment-related toxicities	20 participants	All enrolled subjects will receive therapy with a fixed dose of intravenous relatlimab and nivolumab on days 1 and 29.	Recruiting
NCT04169867	An observational cross-sectional study	Melanoma	To characterize the changes in the gut microbiome by a next-generation sequencing platform and study dietary habit–health correlations	1160 participants	Oncology patients enrolled in the study will be treated with nivolumab, ipilimumab, or atezolizumab as monotherapy or combined treatment.	Unknown status
NCT04136470	An observational cross-sectional study	Melanoma/ NSCLC	To investigate new microbiome-based diagnostics based on sequencing stool samples and determine responders and non-responders to immunotherapy	130 participants	All patients will receive routine treatment, including nivolumab, ipilimumab or atezolizumab as monotherapy or combined treatment.	Unknown status
NCT03643289	An observational prospective study	Melanoma (stage III/IV)	To predict the immunotherapy response based on the gut microbiome composition and examine the relationships between gut microbiome, the presence of tumor infiltrates, and regulatory environments	450 participants	Enrolled patients should be naïve to immunotherapy.	Unknown status
NCT03340129	An interventional Phase II randomized open-label study with parallel assignment	Melanoma (stage IV)	To correlate the gut microbiome with the immunotherapy response and toxicity and assess gastrointestinal integrity along with the bacterial composition in feces, therapeutic response, and irAEs	218 participants	Treatment will consist of nivolumab at 4 doses and then nivolumab every 4 weeks.	Recruiting
NCT05037825	An observational prospective study	Malignant melanoma/ NSCLC/ RCC/ breast cancer	To investigate changes in the relative abundance of microbial taxa from baseline and cycle 2 time points	800 participants	Treatment will consist of a single agent or in combination with another ICI or other treatment agent or modality.	Recruiting
NCT03161756	An interventional Phase I and Phase II non-randomized open-label study with parallel assignment	Melanoma (stage III/IV)	To reveal changes in the gut microbiome for a better understanding of the sensitivity and resistance to the study treatments	72 participants	Arm A will receive nivolumab every 2 weeks and denosumab, and afterward, nivolumab and denosumab every 4 weeks for 24 months (maintenance phase). Arm B will receive ipilimumab + nivolumab every 3 weeks with denosumab. This will be followed by nivolumab and denosumab every 4 weeks for a total of 24 months (maintenance phase).	Unknown status
NCT05877430	An interventional Phase I and Phase II open-label study with sequential assignment	Melanoma/ NSCLC/ HNSCC	To evaluate the effects of different dose levels of CJRB-101 combined with pembrolizumab on the gut microbiome and determine the response, dose-limiting toxicities, and pharmacodynamic effect of the study treatments	160 participants	Patients will be given either a low/high dose of CJRB-101 in combination with pembrolizumab.	Recruiting
NCT04133948	An interventional Phase I and Phase II randomized open-label study with parallel assignment	Malignant melanoma (stage IV)	To evaluate the gut microbiome diversity and its correlation with the pathologic response and toxicities	44 participants	Experimental group A will receive 2 courses of nivolumab pre-surgery. Groups B and C will receive 2 courses of nivolumab + domatinostat pre-surgery. Group D will receive 2 courses of nivolumab + ipilimumab + domatinostat pre-surgery.	Active, not recruiting
NCT06288191	An interventional Phase I and Phase II open-label study with single-group assignment	Cutaneous squamous cell carcinoma	To determine specific gut bacteria and correlate their presence with the treatment response and incidence of treatment-related toxicities	20 participants	Nivolumab and relatlimab will be administered at fixed doses. All patients will receive two doses of nivolumab and relatlimab before surgery on days 1 and 29.	Not yet recruiting

Abbreviations: CTLA-4, cytotoxic T lymphocyte-associated protein 4; HNSCC, head and neck squamous cell carcinoma; ICIs, immune checkpoint inhibitors; IFN-γ, interferon-gamma; mRNA, messenger RNA; OS, overall survival; irAEs, immune-related adverse events; NSCLC, non-small cell lung cancer; PD-1, programmed cell death protein 1; PD-L1, programmed death-ligand 1; PFS, progression-free survival; RCC, renal cell carcinoma.
